# Growth of Ready Meals in Australian Supermarkets: Nutrient Composition, Price and Serving Size

**DOI:** 10.3390/foods10071667

**Published:** 2021-07-20

**Authors:** Katie Wooldridge, Malcolm D. Riley, Gilly A. Hendrie

**Affiliations:** 1 Department of Nutrition and Dietetics, College of Nursing and Health Sciences, Flinders University, Adelaide 5042, Australia; kwooldridge28@gmail.com; 2 Commonwealth Scientific and Industrial Research Organisation (CSIRO) Health and Biosecurity, Adelaide 5000, Australia; Malcolm.Riley@csiro.au

**Keywords:** ready meals, pizza, salads, convenience food, Health Star Rating, sodium, reformulation, food price, serving size, Australian supermarkets

## Abstract

Pre-prepared, or ready meals (frozen, chilled and shelf-stable) are increasingly available in supermarkets in developed countries. This study aimed to investigate how the range of ready meals in Australian supermarkets has changed from 2014 to 2020, and how products vary by price, serving size, nutrient composition and Health Star Rating. Product information was obtained from the FoodTrack™ packaged food database for the years 2014 to 2019 and from an instore audit of products available in Adelaide, Australia for 2020. There was a 13% annual average increase in the number of ready meals available in supermarkets. Serving size did not change (median 350 g, *p*-trend = 0.100) and price increased modestly from 2014 to 2020 (median $1.67 to $1.79/100 g, *p*-trend < 0.001), with chilled ready meals being the most expensive. A modest decrease in sodium density from 2014 to 2020 (median 275 to 240 mg/100 g, *p*-trend < 0.001) was seen. However, the category has a wide range in Health Star Ratings and nutrient composition, highlighting the importance of appropriate consumer choice to optimise health benefits. With the increasing availability of ready meals, global improvements within this category should be encouraged and consumers guided to choose healthier products.

## 1. Introduction

Food choices are the result of complex decisions that, given availability, are largely influenced by taste, cost and convenience [1]. Convenience relates to products that are time or effort saving, and has consequently led to a growth in the convenience food market [2]. Over time, products such as fast food and now pre-prepared meals, have become embedded into the everyday food practices of Western societies [1]. Ready meals can be defined as pre-prepared meals requiring little additional preparation before consumption [3]. Sales data show consistent growth in the category, with Australian ready meal sales experiencing an average annual increase of 8% since 2014 [4]. 

Despite convenience foods becoming a normal part of food intake in the developed world [5], there is a perception that ready meals are lacking in quality, taste and nutrition [5,6,7]. These meals have been targeted for being high in salt and are generally thought to be unhealthy food choices [8,9]. However, there is minimal recent Australian evidence to support this. Research in Europe suggests that ready meals can be a healthier choice, with smaller serving sizes compared to fast food and some homemade recipes, but that the nutritional composition differs widely between products [10,11]. In Australia, some investigators have grouped ready meals with other convenience or ultra-processed foods, such as pizza [12]. Ultra-processed foods are defined as formulations of substances derived from foods and additives [13]. Using the NOVA food classification system, between 93 and 99% of Australian ready meals have been classified as ultra-processed [14,15]. Ultra-processed foods are categorically considered to be unhealthy, and are suggested to be a contributing factor to the obesity epidemic and chronic conditions such as diabetes and cardiovascular disease [13,16]. By grouping almost all ready meals as ultra-processed foods, it is easy to generalise that they are unhealthy. However, studies examining the healthiness of ready meals tend to only use a small sample of meals available in the market [9,15], or focus on one aspect of these products, such as the sodium content [17,18]. 

Sodium is a relevant nutrient in processed foods considering the Australian Government’s commitment to a 30% reduction in population salt intake from 2013 to 2025 [19]. Excess dietary sodium is linked to increased blood pressure and chronic disease risk. The World Health Organisation recommends that adults consume less than 5 g of salt per day [19]. However, literature from 1989 to 2015 suggests that Australian adults are consuming almost double this, at an average of 9.6 g per day [20]. The sodium intake of Australian adults does not appear to have changed in recent decades [20], with ultra-processed foods estimated to contribute approximately half of the adult’s daily sodium intake in the period 2016–2017 [21]. 

To improve the nutritional intake of Australians, the Australian Government introduced the Health Star Rating (HSR) in 2014 and formed the Healthy Food Partnership in 2015 [22,23]. The HSR is a voluntary front-of-pack labelling system that rates the nutritional profile of a product from 0.5 to 5 stars, with 5 stars being a healthier product. The rating is generated by a category-based algorithm which takes into account four components associated with increased risk of chronic disease, being energy, saturated fat, sodium and total sugars per 100 g; as well as positive components of food products including dietary fibre, protein, fruit, vegetables, nuts and legume content. The system aims to provide a quick and easy way for consumers to identify healthier products [22]. The Healthy Food Partnership, on the other hand, is a broader stakeholder approach to improving the dietary health of Australians, and superseded The Australian Food and Health Dialogue which was initiated in 2009 [23]. Product reformulation is a focus of these partnership programs, whereby food manufacturers are encouraged to improve the nutritional composition of their products. This involves setting nutrient targets for specific food categories and working towards these over a period of time. Currently, there is no target for ready meals. However, a proposed target of a maximum 250 mg sodium per 100 g has been deemed both feasible and appropriate for this category [24]. This broadly aligns with other countries such as the United Kingdom, where there is a target of an average 240 mg sodium per 100 g for ready meals by 2024 [25]. Determination of the success of these initiatives relies on regular monitoring and evaluation of the food supply. 

Currently, evaluation of the food supply tends to focus on changes to nutrient composition, in particular sodium, and does not assess changes to other factors such as category size, price or recommended serving size (which in Australia is required on packaging and is determined by the food business). A recent global review encompassing nine countries, including Australia, revealed that sodium content has decreased between 2013 and 2018 for specific food products, including breakfast cereal and bread [26]. Ready meals, however, did not show a reduction over time in the pooled analyses of four studies [26]. These results reflect those seen in the Australian food supply, with ready meals showing no sodium decrease from 2008 to 2017 [17,18]. More recent changes to sodium and other important nutrients in ready meals is unknown.

The growth of the ready meal category over recent years may be associated with other changes that impact on the healthiness of the category. Therefore, the aim of this study was to describe how the range of Australian supermarket ready meals has changed over time from 2014 to 2020 and to explore differences within the category. Factors investigated included the number of products, price, recommended serving size, nutrient composition and Health Star Rating. 

## 2. Materials and Methods

This study examined the availability of ready meals for each year from 2014 to 2020 and compares ready meals to two other food categories—pizzas, and salads. A comparison over time was undertaken for 2014 to 2020 and a cross-sectional analysis for products available instore in 2020. Product data on ready meals, pizzas and salads available in Australian supermarkets were obtained from the FoodTrack™ database (CSIRO and the Heart Foundation, Adelaide, Australia) for the period 2014–2019, and an audit of Adelaide supermarkets in October 2020. Ethical approval was not required for this study. 

Ready meals were defined as supermarket main meals that only require heating before consumption. Products needing additional ingredients or reconstitution (for example with water or milk) were excluded along with meals designed for children, formulated meal replacements, soups, salads, pizzas, baked beans, quiches and products with pastry such as pies and sausage rolls. Frozen, chilled and shelf-stable ready meals were all included. This definition was developed through a combination of those found in the literature [3]. Two other food categories (pizzas and salads) were used as comparator categories for this analysis. These were selected because they were considered to be functionally similar to ready meals (i.e., they are pre-prepared and could function as a meal). Pizzas included all frozen and chilled varieties with toppings, and that required heating only (no addition of extra ingredients). Salads were conceptualized as ready meals that did not require heating. They were defined to have more than three ingredients and included salad kits and bowls, potato and pasta salads and shelf-stable salads such as tuna and bean salads. 

The FoodTrack™ database is an Australian database for packaged food developed by the Commonwealth Scientific and Industrial Research Organisation (CSIRO) and the Heart Foundation. It is updated on an annual cycle using information from Melbourne supermarkets since 2014. Trained data collectors visit Woolworths, Coles, ALDI (since 2016) and IGA (since 2017) supermarkets, and use a customised app with barcode recognition software to collect product information and images of all eligible products for sale [27]. The supermarket and grocery industry in Australia is highly concentrated, with more than 80% of revenue shared between 4 companies—Woolworths 37.4%, Coles 28.4%, ALDI 10.5% and Metcash (owns IGA) 7% [28]. Due to the COVID-19 pandemic in Australia, data collection for FoodTrack™ was suspended for 2020. Therefore, data collection for products in this study was carried out in 2020 in Adelaide supermarkets. Permission to undertake the data collection was obtained by the local or national managers of three conveniently located major supermarkets—a Woolworths, a Coles and an ALDI store. The stores were visited in October 2020 and a set of images was taken of the front of pack, price, nutrition information panel and ingredient list for each of the ready meals, pizzas and salads for sale in the store using a digital camera. Data were transcribed from the images and entered into a Microsoft Excel (2019) spreadsheet by a single investigator (KW). 

Information available for the products over all years included price, number of serves, recommended serving size, nutrients from the nutrition information panel and the Health Star Rating (where provided). Products were categorised as either a ready meal, pizza or salad according to the study definitions. Nutrient values which were recorded on packaging as ‘<1’ were converted to a value of 0.01 and those recorded with a value of ‘<5’ converted to 1. For products with no Health Star Rating or fibre value displayed in the nutrition information panel, these data were recorded as missing values. For the 2020 data collection, storage conditions of products (frozen, chilled or shelf-stable) were also recorded. Outliers were identified using minimum and maximum values and by viewing boxplots. These values were then verified by reviewing the original photographs, and corrected if necessary.

### Statistics

Data analysis was performed using the statistical software package IBM SPSS Statistics version 25. Normality for variables was assessed after plotting histograms and examining z-scores for skewness and kurtosis. Most data distributions were considered non-normal and therefore the distributions of continuous variables, such as price, were described using medians and 25th and 75th percentiles. Non-parametric statistics were generally used to assess differences between categories of ready meals, pizzas and salads. Kruskal–Wallis tests with Bonferroni post hoc comparisons were used to assess evidence for a difference in distributions between years and types of ready meals. The Jonckheere Trend test was used to test linear trend by year. 

Categorical variables, including Health Star Ratings, were described using counts and percentages; and associations between categorical variables examined using the chi-square test. Statistical significance was taken to be a *p*-value of <0.05.

## 3. Results

### 3.1. Change over Time 2014 to 2020

#### 3.1.1. Number of Products

The study data were comprised of 2581 ready meals, 589 pizzas and 610 salads from 2014 to 2020 (Table S1). The 2020 Adelaide data collection consisted of 468 ready meals, 98 pizzas and 133 salads. Each year, the number of products in the ready meal category increased, on average, by 13% compared to 12% for pizzas and 28% for salads. 

#### 3.1.2. Nutrients

**Figure 1:**
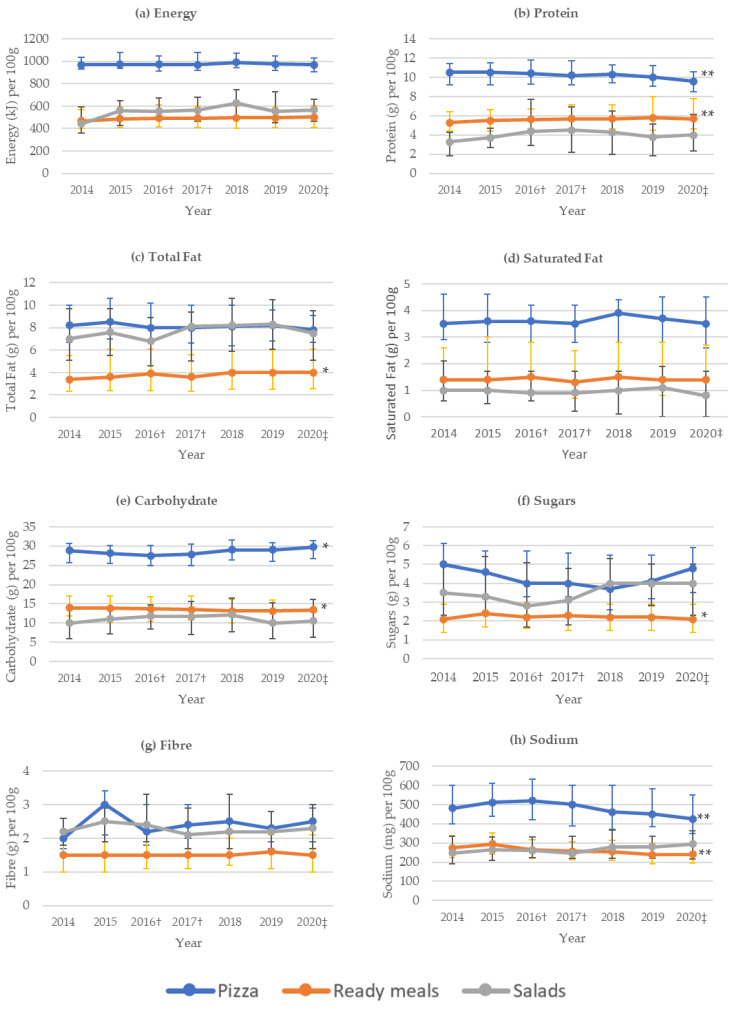
Nutrient content per 100 g for ready meals, pizzas and salads from 2014 to 2020 (median and interquartile range). (**a**) Energy (kJ); (**b**) Protein (g); (**c**) Total Fat (g); (**d**) Saturated Fat (g); (**e**) Carbohydrate (g); (**f**) Sugars (g); (**g**) Fibre (**g**,**h**) Sodium (mg). Data for 2014 to 2019 from FoodTrack™ database; † ALDI products introduced in 2016 and IGA products introduced in 2017; ‡ data for 2020 from Woolworths, Coles and ALDI in Adelaide; * *p*-trend < 0.05; ** *p*-trend < 0.001; §fibre values were available for 1467 (57%) ready meals, 152 (26%) pizzas and 503 (83%) salads.

Figure 1 displays median nutrient content per 100 g for products over each year of the data collection. A statistically significant decrease for ready meals was seen for carbohydrate, sugars and sodium, whereas protein and total fat showed a statistically significant increase over time (*p*-trend < 0.05). Median protein content per 100 g of ready meals increased from 5.3 g in 2014 to 5.7 g in 2020. Median sodium content decreased from 275 mg per 100 g in 2014 to 240 mg in 2020 for ready meals. Over time there was a statistically significant decrease in protein and sodium content for pizzas and an increase in carbohydrate (*p*-trend < 0.05). No trend in nutrient composition was observed for salads from 2014 to 2020.

#### 3.1.3. Display of the Health Star Rating

**Figure 2:**
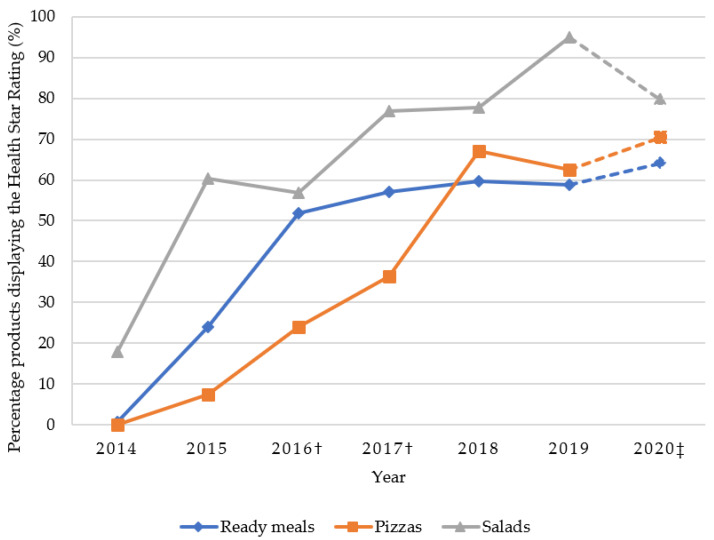
Percentage of ready meals, pizzas and salads displaying the Health Star Rating from 2014 to 2020. Data for 2014 to 2019 from FoodTrack™ database; † ALDI products introduced in 2016 and IGA products introduced in 2017; ‡ data for 2020 from Woolworths, Coles and ALDI in Adelaide.

The percentage of products displaying the Health Star Rating (HSR) is shown in Figure 2. Since 2014, when the labelling system was first introduced in Australia, the percentage of products displaying a rating has increased. From 2016 there has been a plateau for ready meals, with approximately 60% displaying the HSR. A higher percentage of salads have displayed the HSR compared to ready meals and pizzas in all years.

#### 3.1.4. Serving Size and Price

Table 1 shows the median serving sizes and price for products over time. Ready meals had a larger median serving size of 350 g compared to pizzas (113 g) and salads (133 g). The price of ready meals increased from 2014 to 2020 (*p*-trend < 0.001) with median price per 100 g rising from $1.67 in 2014 to $1.79 in 2020. Pizzas did not demonstrate a statistically significant trend in serving size over time, whereas salads showed a statistically significant increase (*p*-trend = 0.032), despite median serving size staying at approximately 133 g. Salads also decreased in price per 100 g (*p*-trend = 0.013) and per serve (*p*-trend = 0.042).

Of the 468 ready meals identified in 2020 in Adelaide, Australia, 228 meals (48.7%) were frozen, 181 (38.7%) chilled and 59 (12.6%) shelf-stable.

Table 2 describes characteristics for frozen, chilled and shelf-stable ready meals. Shelf-stable ready meals had a lower recommended serving size, with a median of 300 g, compared to frozen and chilled meals, with a median of 350 g (*p* = 0.002). Most ready meals were intended to be one serve with 94% of frozen meals being one serve compared to 77% for chilled and 75% for shelf-stable meals.

There was a statistically significant difference in price between all sub-categories of ready meals, whether expressed as total price, price per 100 g or price per recommended serve. Shelf-stable ready meals were the least expensive and chilled ready meals the most expensive, with the total price range being $2.25–$12.00 per single-serve ready meal.

In terms of nutrient content, chilled ready meals had a statistically significant higher protein and saturated fat content per 100 g compared to frozen and shelf-stable meals (*p* < 0.001). Chilled ready meals had the lowest sodium concentration, with a median of 219 mg per 100 g, while shelf-stable meals had the highest, with a median of 300 mg per 100 g.

### 3.2. Differences within the Ready Meal Category in 2020

In 2020, 54.3% of ready meals met the Healthy Food Partnership’s proposed sodium target of a maximum 250 mg per 100 g. The total sodium content of single-serve ready meals ranged from 164 to 3410 mg (more than 20-fold). There was no statistically significant difference for sugar or fibre density between frozen, chilled and shelf-stable meals. The majority of ready meals (64%) displayed the Health Star Rating, ranging from 2 to 5 stars. Most ready meals displayed 3.5 stars (57%). For pizzas, the most frequent rating was 3 stars (48%); and for salads, the most frequent rating was 4 stars (50%). For ready meals, 76% of chilled meals displayed a Health Star Rating compared to 58% and 51% of frozen and shelf-stable meals, respectively. No statistically significant difference between ready meal sub-categories was found for the percentage of products displaying a rating of 3.5 or greater stars.

## 4. Discussion

The rise of convenience foods includes aspects of saving a consumer time, mental and physical effort before, during and after food preparation [29]. Ready meals are an extreme example of convenience foods which are quick to prepare and consume, and easy to clean up following consumption. Over the period studied, there was a strong growth (greater than 10% per year) in the number of different ready meals and pizzas for sale in Australian supermarkets, and very strong growth for pre-prepared salads (more than 25% per year). Chilled ready meals are the fastest-growing ready meal category [4], although in terms of the number of products, in 2020, they were still outnumbered by frozen meals. While suggestive of consumption trends towards quick and easy meal options [6], an increased number of choices of ready meals available does not necessarily reflect increased consumption. 

Nutrient composition of these quick and easy meal options is of interest. Of the three food categories examined, ready meals were nutritionally more similar to salads than to pizza, except for density of total fat and dietary fibre, where salads and pizza were similar. The strong growth in the number of foods in each category means a larger range to choose from, and one basis for choice by consumers could be nutrient composition. Changes to the nutrient composition of a category over time could result from reformulation of foods, or the introduction of new foods with different nutrient composition. Over the seven year observation period, changes in nutrient composition for pizza and ready meals were generally very modest—less than 10% with the exception of sodium where the decrease was 12–13% for pizza and ready meals. If larger changes in dietary intake are required then they may be more easily achieved by switching food categories rather than choosing the better choices within a category, although both strategies could be complementary.

The nutrient content of ready meals differs between countries, although comparison is made with caution. In the United Kingdom, a 2019 survey of 1681 ready meals [30] showed a higher median serve size (400 g compared to 350 g in Australia), with the median energy, total fat, saturated fat and sugar per serve broadly consistent with the difference in serve size. Notably, the median sodium per serve in the UK survey was 130% greater than in the Australian survey.

The finding that sodium content decreased from 2014 to 2020 differs to previous studies that have reported that have reported no change sodium content of Australian ready meals (from 2008 to 2017) [17,18]. Product reformulation is a common means of improving the nutritional quality of the manufactured food supply, but was suggested to have minimal early success in Australia with the Food and Health Dialogue [23]. In July 2020, the Healthy Food Partnership introduced new sodium reformulation targets for 27 food categories [31]. Ready meals are not yet included in these official targets but have a proposed sodium target of a maximum 250 mg per 100 g [31], which remains more than double the definition of a low-sodium food (120 mg per 100 g) [32]. In 2020, almost half of the ready meals included in this study exceeded the proposed target, with some meals containing up to 3400 mg in a single meal. The recommended daily sodium target for adults is less than 2000 mg [19]. Therefore, while the sodium content of ready meals may be declining modestly, there is still substantial room for improvement and the inclusion of ready meals in the Healthy Food Partnership’s targets is warranted. The large range in sodium content of ready meals, and advances in food technology, suggests that a staged reduction in ready meals to a much lower sodium density is achievable [33,34]. Further, consumer demand for healthier alternatives would encourage food manufacturers to participate in voluntary reformulation to achieve meaningful systemic improvements to the food supply [23].

Increasing consumer demand for nutritionally superior food could be addressed by targeted social marketing campaigns or promoting the use by consumers of nutritional signposting such as the front-of-pack Health Star Rating (HSR) [23,35]. While currently voluntary, a mandatory HSR on packaged foods could increase its use [35]. The HSR ratings of ready meals ranged from 2 to the maximum 5 stars in 2020, demonstrating the large range in the nutritional quality of products available, although the number of products at each extreme was small. However, only 64% of ready meals displayed the rating in 2020, so assessment was not available for more than one third of ready meal products for sale. Researchers and practitioners have often used a cutoff of 3.5 stars or greater to indicate a healthier food [12,14,15], because this cutoff aligns best with recommendations of the Australian Dietary Guidelines [36]. The majority of ready meals (83.6%) displayed 3.5 or greater stars, which suggests a generally healthy option, especially compared to other convenience products such as pizza (40.5% with 3.5 stars or greater). Based on these results, the assignment of the entire category of ready meals as unhealthy [7] seems unwarranted. 

The HSR is only one way of assessing the nutritional quality of food products [12], and has been criticised for promoting discretionary food choices [37]. A combined approach has therefore been adopted in several studies, whereby the health benefit of a food is evaluated using different methods such as the HSR and classifications used in the Australian Dietary Guidelines [12,14,15]. For ready meals, one Australian study focusing on a limited range of chilled ready meals found Woolworths home-brand ready meals to have a higher proportion of healthier products compared to other convenience foods, such as pizzas [15]. This assessment was made using the HSR, Australian Dietary Guidelines and the NOVA food classification system. Coles home-brand ready meals, on the other hand, had a higher proportion of unhealthy products which highlights the importance of the manufacturer in determining the nutritional quality of their product range [15].

Price and value for money form part of consumers’ purchase decision when buying convenience foods [38]. The present study found that the price of ready meals has increased over time at an average rate of 1.6% per year, the same as the increase in the Australian consumer price index from 2014 to 2019 [39]. The affordability of ready meals was therefore unchanged over the period of this study, whereas pizzas and salads have become more affordable in relative terms. There was also a large range in the price of ready meals, suggesting that price points meet a range of budgets. As an example, in 2020, one single-serve lasagne ready meal cost $2.25 compared to $12.00 for a beef steak single-serve ready meal. Based on price per serve, pizzas were generally the least expensive, while ready meals were the most expensive. However, this comparison may be inappropriate, because it is not clear whether the different food categories are comparable in their use. The median stated serving size for ready meals of 350 g is substantially larger than that for pizzas and salads, which might suggest that the latter are more commonly used as a component of a meal, or consumed in more than one stated serve. For packaged food in Australia, the suggested serving size is a required component of the nutrition information panel and is determined by the manufacturer [40]. Future research into how ready meals and other convenience foods are consumed by different population segments would help in understanding how they contribute to nutritional health.

Ready meals can be well suited for the life stage and situation of some individuals. In a recent study based in the United Kingdom, higher consumption of ready meals was seen in 18 to 24 year olds and in males after adjustment for potential confounders [41]. Higher consumption in younger adults is supported by findings that younger adults tend to hold a more positive attitude towards convenience foods compared to the elderly [2,42]. Older generations generally prefer traditional cooking and have therefore been found to be less accepting of ready meals compared to younger adults [2,42]. Younger adults may also live independently and have limited cooking skills, and therefore seek easily prepared meals that serve one person, such as a ready meal. Ready meals are also recognised as being a suitable food product for elderly people who may be unable to cook for themselves but still wish to maintain their independence [42,43]. The large range in ready meals available ensures that consumers can choose meals suitable for their preferences and needs (including nutritional needs). 

### Strengths and Limitations

A strength of this study was the use of the FoodTrack™ database, which captures more than 90% of packaged foods sold in Australian supermarkets with annual data from 2014 to 2019. Prior to 2016, products from ALDI were not included in the database and prior to 2017, IGA products were not included. The total number of products available each year is likely to have been influenced by the addition of these product sources, but there is no discontinuity in the time trends of median values for nutrient composition. One limitation to this study was the use of 2020 data collected from Adelaide supermarkets, whereas FoodTrack™ data were routinely collected from Melbourne supermarkets. The FoodTrack™ data collection did not occur in 2020. However, Australian supermarket businesses have national distribution networks, and the use of the Adelaide data allowed an additional contemporary data point and extra data collection such as ready meal sub-categories based on storage requirements. A further limitation to this study is that packaged foods sold exclusively online were not included. This currently small route to market may provide nutritionally distinctive packaged foods. However, this remains to be explored.

The definitions of ready meals vary widely in the literature [6]. This study used a clear definition which was easy to operationalise, but the categorisation here may be different to what are considered ready meals by others. For example, soups were excluded from the definition for this study but may be considered a ready meal (i.e., ‘heat and eat’) by others [44]. By comparing ready meals to pizzas and salads, differences within the broader convenience meal category have become apparent. All convenience meals are not the same, and research on other specific convenience food categories is likely to be insightful. It is important that clear, pragmatic and robust definitions are applied when describing these categories in order for studies to be repeatable, and for results to be readily actionable. 

By considering the eight nutrients that are required on Australian food labels, this work has built on previous research which has only examined the sodium content of ready meals [17,18]. In Australia, displaying the fibre content is not mandatory unless a claim is made about fibre content or function on the food label [40], and therefore the results on fibre are limited to only those products displaying fibre in the nutrient information panel. These foods may have been higher in fibre relative to those that did not display a fibre value. Other nutrients not displayed on the nutrient information panel were also not investigated. Similarly, the HSR was only considered for examined products which displayed the rating. While the uptake of the HSR in convenience foods is better than the average for uptake of the HSR [45], the percentage of ready meals that displayed the rating has not risen above 65%. Products displaying the HSR reportedly have a higher HSR than those not displaying the rating [45], and therefore an assessment of change in HSR that relies on the displayed rating is incomplete. The use of HSR on food labels appears to be clearly in the public interest, and some food companies have chosen to display the HSR on all of their products. 

Finally, this study only assessed availability of selected supermarket convenience foods (i.e., what was on the supermarket shelf) and not consumption of these foods. It is not known what specific foods are the most popular and how people choose and consume ready meals. For example, are ready meals generally consumed on their own or with additional ingredients? Do people generally consume the recommended serving size, and what proportion of purchased food is wasted? While the marketplace choice is changing, an understanding of factors such as these is necessary to assess the role of ready meals and other convenience foods in the provision of healthy food intake.

## 5. Conclusions

The ready meals category has rapidly grown in Australia, as have pre-prepared salads, another convenience food category. Ready meals include many hundreds of different foods which vary substantially in terms of price and nutritional quality. Healthier ready meals are available, and the majority of ready meals carry the Health Star Rating as a guide for consumers. A modest trend towards lower-sodium ready meals is evident, although some meals are still very high in sodium. The inclusion of ready meals as part of the Australian Healthy Food Partnership’s reformulation program may accelerate changes in nutrition composition. Future research is also needed to understand how ready meals and other convenience foods fit into the total diet and what factors are motivating their purchase. 

## Figures and Tables

**Table 1:** Serving size and price of ready meals, pizzas and salads from 2014 to 2020 (median).

	Type of Product	Year Data Were Collected							*p*-Value §	*p*-Value for Trend *
		2014	2015	2016 †	2017 †	2018	2019	2020 ‡		
		Median								
Serving size (g)	Ready meals	350	340 ^a^	350 ^b^	350	350	350 ^a,b^	350	0.006	0.100
	Pizzas	100	106	113	113	115	118	104	0.402	0.090
	Salads	133	133	140	131	133	125	133	0.185	0.032
Total price of product ($)	Ready meals	5.80 ^a,b^	6.25 ^c^	6.30 ^d^	6.00 ^e,f^	6.50	6.85 ^a,c,d,e^	6.51 ^b,f^	<0.001	<0.001
	Pizzas	6.96	6.99	6.50	6.50	5.00	6.50	6.75	0.033	0.467
	Salads	5.69 ^a,b,c^	5.84 ^d,e^	4.29 ^a,d,f,g^	4.00 ^b,e,h,i^	4.99 ^c^	5.00 ^f,h^	5.00 ^g,i^	<0.001	0.488
Price per 100 g ($)	Ready meals	1.67 ^a,b,c^	1.81	1.76	1.75	1.76 ^a^	1.80 ^b^	1.79 ^c^	0.002	<0.001
	Pizzas	1.75 ^a,b^	1.74	1.3 ^a^	1.30	1.30 ^b^	1.35	1.40	0.010	0.066
	Salads	1.84	1.83	2.00	1.59	1.56	1.43	1.67	0.090	0.013
Price per serve ($)	Ready meals	5.49 ^a,b,c^	5.69 ^d^	5.00 ^e,f^	5.70 ^g^	6.00 ^a^	6.30 ^b,c,d,e,g^	5.90 ^c,f^	<0.001	<0.001
	Pizzas	1.75	1.75	1.25	1.25	1.25	1.63	1.69	0.246	0.918
	Salads	2.32	2.31	2.75	2.00	2.00	1.66	2.50	0.099	0.042

Data for 2014 to 2019 from FoodTrack™ database; † ALDI products introduced in 2016 and IGA products introduced in 2017; ‡ data for 2020 from Woolworths, Coles and ALDI in Adelaide; § Kruskal–Wallis tests; values for years within a row with like superscript letters significantly different using the Bonferroni post hoc test (*p* = 0.05); * Jonckheere Trend tests.

**Table 2:** Serving size, number of serves, price, nutrient content and Health Star Rating of ready meals by storage classification in 2020 (median, count and percentage).

Ready Meals 2020	Storage (Ready Meals)			Total (*n* = 468)	*p*-Value
	Frozen (*n* = 228)	Chilled (*n* = 181)	Shelf-Stable (*n* = 59)		
	Median				
Serving size (g) †	350 ^a^	350 ^b^	300 ^a,b^	350	0.002
One serve (*n*, %) ‡	214 (93.9)	139 (76.8)	44 (74.6)	397 (84.8)	<0.001
**Price** †					
Total price of product ($)	5.60 ^a^	8.83 ^a^	3.00 ^a^	6.51	<0.001
Price per 100 g ($)	1.60 ^a^	2.14 ^a^	1.14 ^a^	1.79	<0.001
Price per serve ($)	4.80 ^a^	7.49 ^a^	3.29 ^a^	5.90	<0.001
**Nutrients per 100 g** †					
Energy (kJ)	472 ^a,b^	525 ^a^	581 ^b^	503	<0.001
Protein (g)	5.3 ^a^	7.3 ^a,b^	4.9 ^b^	5.7	<0.001
Total fat (g)	3.2 ^a^	4.6 ^a^	3.8	4.0	<0.001
Saturated fat (g)	1.3 ^a^	2 ^a,b^	1.2 ^b^	1.4	<0.001
Carbohydrate (g)	13.7 ^a^	12.5 ^a,b^	17.9 ^b^	13.4	<0.001
Sugar (g)	2.1	2.0	2.2	2.1	0.447
Fibre (g)	1.4	1.5	1.6	1.5	0.122
Sodium (mg)	248 ^a^	219 ^a^	300 ^a^	240	<0.001
**Nutrients per serve** †					
Energy (kJ)	1544 ^a^	1772 ^a^	1733	1649	<0.001
Protein (g)	18.3 ^a^	24.8 ^a^	15.2 ^a^	19.16	<0.001
Total fat (g)	10.4 ^a^	15.2 ^a,b^	10.8 ^b^	12.73	<0.001
Saturated fat (g)	4.16 ^a^	6.65 ^a,b^	3.28 ^b^	4.80	<0.001
Total carbohydrate (g)	45.1 ^a^	41.7 ^a^	47.6	44.45	0.017
Sugar (g)	7.02	6.60	5.60	6.89	0.458
Fibre (g)	4.50	5.00	4.46	4.86	0.413
Sodium (mg)	805 ^a^	700 ^a^	812	784	0.003
Fibre displayed (*n*, %) ‡	115 (50.4)	153 (84.5)	46 (78)	314 (67.1)	<0.001
**Health Star Rating** ‡					
Displaying Health Star Rating (*n*, %)	133 (58.3)	137 (75.7)	30 (50.8)	300 (64.1)	<0.001
Health Star Rating ≥ 3.5 stars (*n*, %) §	107 (80.5)	116 (84.7)	24 (80)	247 (82.3)	0.622

† Kruskal–Wallis tests; category values within a row with like superscript letters were significantly different using the Bonferroni post hoc test (*p* = 0.05); ‡ chi-square tests; § including only products displaying the Health Star Rating (*n* = 300).

## Data Availability

The FoodTrack™ data presented in this study are not publicly available for commercial reasons. The 2020 Adelaide data are available on request from the corresponding author.

## Supplementary Materials

The following are available online at https://www.mdpi.com/article/10.3390/foods10071667/s1, Table S1: Number of ready meals, pizzas and salads collected each year and percentage change from 2014 to 2020; Table S2: Serving size, price and nutrient content of ready meals, pizzas and salads from 2014 to 2020 (25th and 75th percentile); Table S3: Nutrient content of ready meals, pizzas and salads from 2014 to 2020 (median); Table S4: Health Star Rating of ready meals, pizzas and salads displaying the rating from 2014 to 2020.

## References

[1] Drewnowski A., Monsivais P., Marriott B.P., Birt D.F., Stallings V.A., Yates A.A. (2020). Chapter 10—Taste, cost, convenience, and food choices. Present Knowledge in Nutrition.

[2] Barska A. (2017). Millennial consumers in the convenience food market. Management.

[3] Jackson P., Viehoff V. (2016). Reframing convenience food. Appetite.

[4] Euromonitor International (2019). Ready meals in Australia.

[5] Halkier B. (2017). Normalising convenience food?. Food Cult. Soc..

[6] Jackson P. (2018). Reframing Convenience Food.

[7] Laguna L., Gómez B., Garrido M.D., Fiszman S., Tarrega A., Linares M.B. (2020). Do consumers change their perception of liking, expected satiety, and healthiness of a product if they know it is a ready-to eat meal?. Foods.

[8] VicHealth Australian Ready Meals Are Saltier than Ever. https://www.vichealth.vic.gov.au/media-and-resources/media-releases/australian-ready-meals-are-saltier-than-ever.

[9] LiveLighter An Inconvenient Truth: Health Experts Reveal Ready Meals not so Convenient for Good Health. https://livelighter.com.au/news/An-inconvenient-truth-Health-experts-reveal-ready-meals-not-so-convenient-for-good-health.

[10] Trattner C., Elsweiler D., Howard S. (2017). Estimating the healthiness of internet recipes: A cross-sectional study. Front. Public Health.

[11] Blackham T., Abayomi J., Davies I. (2012). Comparison of the nutritional quality of Indian takeaway and supermarket ready meals. Proc. Nutr. Soc..

[12] Crino M., Sacks G., Dunford E., Trieu K., Webster J., Vandevijvere S., Swinburn B., Wu J.Y., Neal B. (2018). Measuring the healthiness of the packaged food supply in Australia. Nutrients.

[13] Monteiro C.A., Cannon G., Levy R.B., Moubarac J.-C., Louzada M.L.C., Rauber F., Khandpur N., Cediel G., Neri D., Martinez-Steele E. (2019). Ultra-processed foods: What they are and how to identify them. Public Health Nutr..

[14] The George Institute for Global Health (2020). FoodSwitch: State of the Food Supply.

[15] Pulker C.E., Farquhar H.R., Pollard C.M., Scott J.A. (2020). The nutritional quality of supermarket own brand chilled convenience foods: An Australian cross-sectional study reveals limitations of the Health Star Rating. Public Health Nutr..

[16] Elizabeth L., Machado P., Zinöcker M., Baker P., Lawrence M. (2020). Ultra-processed foods and health outcomes: A narrative review. Nutrients.

[17] Christoforou A.K.M., Dunford E.K.M.P.H., Neal B.C.P. (2013). Changes in the sodium content of Australian ready meals between 2008 and 2011. Asia Pac. J. Clin. Nutr..

[18] Farrand C., Santos J. (2017). Changes in Salt Levels in Ready Meals, Australia (2010–2017).

[19] World Health Organisation Salt Reduction. https://www.who.int/news-room/fact-sheets/detail/salt-reduction.

[20] Land M.-A., Neal B.C., Johnson C., Nowson C.A., Margerison C., Petersen K.S. (2018). Salt consumption by Australian adults: A systematic review and meta-analysis. Med. J. Aust..

[21] Bolton K.A., Webster J., Dunford E.K., Jan S., Woodward M., Bolam B., Neal B., Trieu K., Reimers J., Armstrong S. (2020). Sources of dietary sodium and implications for a statewide salt reduction initiative in Victoria, Australia. Brit. J. Nutr..

[22] Australian Government Health Star Rating System. http://healthstarrating.gov.au/internet/healthstarrating/publishing.nsf/Content/home.

[23] Jones A., Magnusson R., Swinburn B., Webster J., Wood A., Sacks G., Neal B. (2016). Designing a Healthy Food Partnership: Lessons from the Australian Food and Health Dialogue. BMC Public Health.

[24] Rosewarne E., Huang L., Farrand C., Coyle D., Pettigrew S., Jones A., Moore M., Webster J. (2020). Assessing the Healthy Food Partnership’s proposed nutrient reformulation targets for foods and beverages in Australia. Nutrients.

[25] Nicholas J., Knowles B., Rayson Z. (2020). Salt Reduction Targets for 2024.

[26] Santos J.A., Sparks E., Thout S.R., McKenzie B., Trieu K., Hoek A., Johnson C., McLean R., Arcand J., Campbell N.R.C. (2019). The science of salt: A global review on changes in sodium levels in foods. J. Clin. Hypertens..

[27] CSIRO FoodTrack™ Food and Nutrient Database. https://www.csiro.au/en/Research/Health/Nutrition-science/Nutrition-databases/FoodTrack.

[28] Youl T. G4111: Supermarkets and Grocery Stores in Australia; IbisWorld Report. https://www.ibisworld.com/au/industry/supermarkets-grocery-stores/1834/.

[29] AlOudat M., Magyar N., Simon-Sakardi L., Lugasi A. (2021). Nutritional content of ready-to-eat meals in groceries in Hungary. Int. J. Gast Food Sci..

[30] Hillier S.E., Nunn O., Lorrain-Smith K. (2020). An analysis of the nutritional value of UK supermarket ready meals. Proc. Nutr. Soc..

[31] Australian Government Department of Health Welcome to the Healthy Food Partnership Website. https://www.health.gov.au/initiatives-and-programs/healthy-food-partnership.

[32] Heart Foundation Is Salt Bad for Your Heart?. https://www.heartfoundation.org.au/Heart-health-education/Salt-and-heart-health.

[33] Mitchell M., Brutnon N.P., Fitzgerald R.J., Wilkinson M.G. (2013). The use of herbs, spices, and whey proteins as natural flavor enhancers and their effect on the sensory acceptability of reduced-salt chilled ready-meals. J. Culin. Sci. Tech..

[34] Scrinis G., Monteiro C.A. (2018). Ultra-processed foods and the limits of product reformulation. Public Health Nutr..

[35] Bablani L., Ni Mhurchu C., Neal B., Skeels C.L., Staub K.E., Blakely T. (2020). The impact of voluntary front-of-pack nutrition labelling on packaged food reformulation: A difference-in-differences analysis of the Australasian Health Star Rating scheme. PLoS Med..

[36] Jones A., Rådholm K., Neal B. (2018). Defining ‘unhealthy’: A systematic analysis of alignment between the Australian Dietary Guidelines and the Health Star Rating system. Nutrients.

[37] Lawrence M.A., Dickie S., Woods J.L. (2018). Do nutrient-based front-of-pack labelling schemes support or undermine food-based dietary guideline recommendations? Lessons from the Australian Health Star Rating system. Nutrients.

[38] Horning M.L., Fulkerson J.A., Friend S.E., Story M. (2017). Reasons parents buy prepackaged, processed meals: It is more complicated than “I don’t have time”. J. Nutr. Educ. Behav..

[39] Australian Bureau of Statistics Tables 1 and 2. CPI: All Groups, Index Numbers and Percentage Changes [Time Series Spreadsheet]. https://www.abs.gov.au/statistics/economy/price-indexes-and-inflation/consumer-price-index-australia/latest-release.

[40] Food Standards Australia New Zealand (2013). Nutrition Information: User Guide to Standard 1.2.8—Nutrition Information Requirements.

[41] Birch J., Petty R., Hooper L., Bauld L., Rosenberg G., Vohra J. (2019). Clustering of behavioural risk factors for health in UK adults in 2016: A cross-sectional survey. J. Public Health.

[42] Peura-Kapanen L., Jallinoja P., Kaarakainen M. (2017). Acceptability of convenience food among older people. SAGE Open.

[43] Hoffman R. (2017). Micronutrient deficiencies in the elderly—could ready meals be part of the solution?. J. Nutr. Sci..

[44] Kim S., Kim S. (2020). Recent surge of ready meals in South Korea: Can they be healthy alternatives?. Public Health Nutr..

[45] Shahid M., Neal B., Jones A. (2020). Uptake of Australia’s Health Star Rating system 2014–2019. Nutrients.

