# Supplementary material for: Growth of Ready Meals in Australian Supermarkets: Nutrient Composition, Price and Serving Size
Source: Foods. 2021 Jul 20;10(7):1667. doi: 10.3390/foods10071667 (PMC8304220; doi:10.3390/foods10071667)
Supplement: Supplementary file 1 [file foods-10-01667-s001.zip › foods-1262038-supplementary.pdf]

nutrients

MDPI

Supplementary Materials

Table S1. Number of ready meals, pizzas and salads collected each year and percentage change from 2014 to 2020.

Type of product	Year							Total	Percentage increase 2014 to 2020	Average percentage change for each year
	2014	2015	2016†	2017†	2018	2019	2020‡			
Ready meals	243	241	384	406	389	450	468	2581	93%	13%
Pizzas	60	54	92	107	79	99	98	589	63%	12%
Salads	39	48	95	82	112	101	133	610	241%	28%
Total	342	344	571	596	580	650	699	3782	104%	15%

Data for 2014 to 2019 from FoodTrack™ database; †ALDI products introduced in 2016 and IGA products introduced in 2017; ‡Data for 2020 from Woolworths, Coles and ALDI in Adelaide.

Table S2: Serving size, price and nutrient content of ready meals, pizzas and salads from 2014 to 2020 (25th and 75th percentile).

	Type of product	2014		2015		2016†		2017†		2018		2019		2020‡		Total	
		25th	75th	25th	75th	25th	75th	25th	75th	25th	75th	25th	75th	25th	75th	25th	75th
Serving size (g)	Ready meals	300	360	280	370	285	360	300	370	300	360	305	375	300	375	300	370
	Pizzas	78	135	80	125	63	125	67	125	75	135	85	150	80	125	78	125
	Salads	125	150	125	188	116	190	110	185	123	185	100	180	117	185	116	185
**Price**																	
Total price of product ($)	Ready meals	4.20	6.99	4.50	7.00	4.00	7.00	4.00	7.00	4.00	8.00	4.99	8.50	4.00	8.52	4.20	7.99
	Pizzas	5.25	7.50	4.99	7.50	4.00	7.25	4.00	7.50	3.99	7.00	3.99	7.50	4.50	7.50	4.00	7.50
	Salads	5.00	6.98	5.00	6.00	3.49	5.00	3.50	5.00	4.00	6.00	4.50	6.00	4.00	5.99	4.00	5.99
Price per 100 g ($)	Ready meals	1.20	1.91	1.19	2.00	1.14	2.00	1.15	2.03	1.05	2.19	1.25	2.14	1.17	2.14	1.15	2.06
	Pizzas	1.29	2.15	1.17	2.11	1.01	1.81	0.98	1.75	0.96	1.80	1.06	2.00	1.06	1.91	1.06	1.92
	Salads	1.42	2.62	1.49	2.33	1.42	2.26	1.25	2.25	1.25	2.35	1.20	2.27	1.25	2.29	1.25	2.28
Price per serve ($)	Ready meals	3.99	6.49	3.55	6.69	3.50	6.99	3.99	6.99	3.50	7.50	4.00	8.00	3.50	8.01	3.50	7.00
	Pizzas	1.15	2.50	0.88	1.99	0.75	1.75	0.75	2.00	0.74	2.25	0.76	3.25	0.83	2.50	0.75	2.00
	Salads	1.66	5.00	1.66	5.00	1.89	3.99	1.50	3.68	1.49	4.00	1.25	3.99	1.49	4.00	1.50	4.00
**Nutrient per 100 g**																	
Energy (kJ)	Ready meals	400	569	408	592	412	609	409	599	400	598	411	588	411	608	408	597
	Pizzas	928	1035	934	1080	910	1050	920	1080	941	1070	921	1050	936	1030	928	1050
	Salads	362	595	429	650	474	672	462	680	491	746	451	731	461	663	457	684
Protein (g)	Ready meals	4.4	6.4	4.4	6.6	4.6	6.7	4.7	7.1	4.6	7.1	4.5	8.0	4.6	7.8	4.6	7.1
	Pizzas	9.2	11.4	9.2	11.5	9.3	11.8	9.2	11.7	9.4	11.3	9.1	11.2	8.5	10.6	9.1	11.2
	Salads	1.8	4.3	2.7	4.7	2.9	7.7	2.2	6.9	2.0	6.5	1.8	5.1	2.3	6.1	2.1	6.2
Total fat (g)	Ready meals	2.3	5.5	2.4	6.1	2.4	6.1	2.3	5.6	2.5	5.9	2.5	6.0	2.6	6.1	2.4	5.9
	Pizzas	6.7	10.0	7.0	10.6	6.9	10.2	6.6	10.0	6.4	10.0	6.8	9.6	6.7	9.1	6.8	10.0
	Salads	5.1	9.7	5.5	9.7	4.6	8.9	5.0	9.4	5.9	10.6	6.1	10.5	5.1	9.5	5.3	9.7

Saturated fat (g)	Ready meals	0.9	2.6	0.9	3.0	0.9	2.8	0.7	2.5	0.9	2.8	0.8	2.8	0.9	2.7	0.8	2.8
	Pizzas	2.9	4.6	2.8	4.6	2.8	4.2	2.8	4.2	2.8	4.4	2.8	4.5	2.6	4.5	2.8	4.4
	Salads	0.6	2.1	0.5	1.7	0.6	1.7	0.2	1.7	0.1	1.7	0.0	1.9	0.0	1.7	0.3	1.7
Carbohydrate (g)	Ready meals	11.8	17.0	10.0	17.0	10.5	16.8	10.8	17.0	10.0	16.1	9.9	15.9	10.3	16.1	10.4	16.4
	Pizzas	25.7	30.6	25.4	30.2	24.9	30.1	25.0	30.4	26.4	31.5	26.0	30.9	26.7	31.3	25.6	30.5
	Salads	6.0	13.9	7.2	14.1	8.5	14.8	7.0	15.7	7.8	16.5	6.0	15.3	6.4	16.1	6.9	15.7
Sugars (g)	Ready meals	1.4	2.9	1.7	3.2	1.6	3.0	1.5	3.1	1.5	2.9	1.5	2.9	1.4	2.9	1.5	3.0
	Pizzas	3.4	6.1	3.3	5.7	3.3	5.7	3.3	5.6	2.6	5.5	3.2	5.5	3.5	5.9	3.3	5.7
	Salads	2.3	5.1	2.5	5.4	1.7	5.1	1.8	4.8	2.2	5.3	2.8	5.0	2.3	5.0	2.2	5.0
Fibre (g)	Ready meals	1.0	1.8	1.0	1.9	1.1	2.2	1.1	2.1	1.2	2.0	1.1	2.1	1.0	2.1	1.1	2.0
	Pizzas	1.7	2.2	2.1	3.4	1.8	3.0	2.0	3.0	2.0	3.3	1.9	2.8	1.9	2.9	1.9	3.0
	Salads	1.8	2.6	1.9	2.9	1.9	3.3	1.7	2.9	1.7	3.3	1.7	2.8	1.7	3.0	1.7	2.9
Sodium (mg)	Ready meals	225	339	240	352	220	317	213	305	210	313	190	300	195	300	210	316
	Pizzas	400	601	440	610	420	630	388	600	370	600	384	580	350	550	390	595
	Salads	190	333	208	330	222	331	220	336	221	368	220	336	215	362	220	352
**Nutrient per serve**																	
Energy (kJ)	Ready meals	1229	1992	1220	1996	1222	2093	1267	2055	1264	2044	1340	2052	1280	2104	1269	2055
	Pizzas	743	1297	872	1234	640	1199	792	1236	690	1363	872	1450	802	1205	782	1250
	Salads	481	1015	613	1009	582	1171	557	1171	712	1188	482	1103	576	1085	571	1110
Protein (g)	Ready meals	13.4	21.6	13.5	22.4	14.2	22.8	14.9	24.2	14.3	25.2	14.7	27.9	14.6	26.5	14.4	24.5
	Pizzas	8.5	15.5	8.2	15.8	7.2	14.2	7.2	14.5	7.5	16.3	7.7	16.5	7.4	13.6	7.5	14.8
	Salads	1.7	7.3	3.2	8.5	3.3	12.8	2.7	11.6	2.7	10.7	2.1	8.1	2.8	12.4	2.7	10.5
Total fat (g)	Ready meals	7.4	17.6	7.4	18.8	7.5	19.4	7.2	17.9	8.0	18.6	8.0	20.7	8.1	19.6	7.7	19.0
	Pizzas	6.1	11.3	6.5	11.0	5.3	10.9	5.5	11.0	5.7	11.3	5.9	13.0	5.7	10.9	5.9	11.2
	Salads	6.8	15.6	7.6	15.1	6.4	14.2	6.4	14.0	7.6	15.4	7.8	16.0	7.0	13.6	6.9	14.8
Saturated fat (g)	Ready meals	2.8	8.3	2.7	8.8	2.7	9.0	2.4	8.1	2.7	8.8	2.7	9.3	2.6	8.4	2.6	8.6

	Pizzas	2.8	5.0	3.2	5.0	2.3	4.8	2.6	4.9	2.6	5.5	2.6	5.8	2.5	5.4	2.6	5.1
	Salads	0.7	3.2	0.5	2.3	0.7	2.3	0.3	2.1	0.1	2.1	0.0	2.5	0.0	2.1	0.5	2.3
Carbohydrate (g)	Ready meals	34.9	58.8	31.3	56.6	31.3	57.8	33.6	57.1	30.0	56.2	31.6	56.2	32.0	56.4	32.0	56.6
	Pizzas	19.0	41.0	20.0	36.9	19.2	34.7	20.8	37.8	19.5	43.9	26.7	43.3	22.4	38.4	20.9	38.5
	Salads	7.5	24.3	9.1	27.4	11.5	26.2	10.0	26.3	10.4	25.7	8.4	23.3	9.6	24.8	10.0	25.3
Sugars (g)	Ready meals	4.5	10.0	4.9	10.2	4.8	9.8	4.8	10.1	4.8	9.8	4.8	10.4	4.6	10.0	4.8	10.0
	Pizzas	3.1	8.4	3.3	7.7	2.6	6.3	3.0	6.4	2.5	6.8	3.4	6.9	3.8	7.5	3.1	6.9
	Salads	3.3	7.0	3.4	8.3	2.5	6.4	2.3	6.3	2.8	7.3	3.7	7.2	3.3	6.7	3.0	7.0
Fibre (g)	Ready meals	3.2	6.1	3.0	6.1	3.5	6.6	3.4	7.0	3.5	6.5	3.5	6.8	3.2	6.7	3.4	6.7
	Pizzas	0.9	2.3	1.6	3.9	1.9	3.4	1.9	3.5	1.8	3.5	1.4	2.6	2.1	3.5	1.8	3.4
	Salads	2.4	3.5	2.7	4.5	2.8	5.6	2.1	4.5	2.2	5.5	2.1	3.6	2.2	4.3	2.3	4.4
Sodium (mg)	Ready meals	718	1085	721	1087	669	1030	669	1008	665	1015	630	1001	633	965	665	1031
	Pizzas	338	689	349	844	350	731	349	763	348	850	371	739	281	752	342	752
	Salads	266	487	307	494	287	629	270	513	286	572	263	513	276	581	278	546

Data for 2014 to 2019 from FoodTrack™ database; †ALDI products introduced in 2016 and IGA products introduced in 2017; ‡Data for 2020 from Woolworths, Coles and ALDI in Adelaide.

Table S3: Nutrient content of ready meals, pizzas and salads from 2014 to 2020 (median).

	Type of product	2014	2015	2016†	2017†	2018	2019	2020‡	Total	*p*-value§	*p*-value for trend*
		Year data was collected<br>Median									
**Nutrient per 100 g**											
Energy (kJ)	Ready meals	468	488	494	492	495	496	503	495	0.324	0.068
	Pizzas	968	973	973	970	990	976	969	974	0.841	0.804
	Salads	443[a]	560	554	565	629[a]	554	567	567	0.006	0.117
Protein (g)	Ready meals	5.3[a,b]	5.5	5.6	5.7	5.7	5.8[a]	5.7[b]	5.6	0.004	< 0.001
	Pizzas	10.45[a]	10.5[b]	10.4[c]	10.2[d]	10.3[e]	10.0	9.6[a,b,c,d,e]	10.2	0.005	< 0.001
	Salads	3.3[a,b]	3.7	4.4[a]	4.5[b]	4.3	3.8	4.0	4.0	0.002	0.911
Total fat (g)	Ready meals	3.4	3.6	3.9	3.6	4.0	4.0	4.0	3.8	0.027	0.004
	Pizzas	8.2	8.5	8.0	8.0	8.1	8.2	7.8	8.0	0.767	0.153
	Salads	7.0	7.6	6.8	8.1	8.2	8.3	7.5	7.8	0.066	0.165
Saturated fat (g)	Ready meals	1.4	1.4	1.5	1.3	1.5	1.4	1.4	1.4	0.409	0.648
	Pizzas	3.5	3.6	3.6	3.5	3.9	3.7	3.5	3.6	0.791	0.776
	Salads	1.0	1.0	0.9	0.9	1.0	1.1	0.8	1.0	0.735	0.180
Carbohydrate (g)	Ready meals	14[a]	13.9	13.7	13.5	13.2	13.2[a]	13.4	13.5	0.009	0.002
	Pizzas	28.8	28.1	27.5[a]	27.8	29.0	29.0	29.7[a]	28.5	0.005	0.001
	Salads	10.0	11.1	11.8	11.7	12.2	10.0	10.6	11.5	0.608	0.878
Sugars (g)	Ready meals	2.1	2.4	2.2	2.3	2.2	2.2	2.1	2.2	0.063	0.028
	Pizzas	5.0	4.6	4.0	4.0	3.7	4.1	4.8	4.3	0.114	0.874
	Salads	3.5	3.3	2.8[a]	3.1	4.0	4[a]	4.0	3.6	0.074	0.088
Fibre (g)	Ready meals	1.5	1.5	1.5	1.5	1.5	1.6	1.5	1.5	0.444	0.927
	Pizzas	2.0	3.0	2.2	2.4	2.5	2.3	2.5	2.4	0.227	0.686
	Salads	2.2	2.5	2.4	2.1	2.2	2.2	2.3	2.2	0.419	0.372

Sodium (mg)	Ready meals	275[a,b,c]	295[d,e,f,g,h]	263[d,i,j]	256[a,e]	255[f]	239[b,g,i]	240[c,h,j]	259	<0.001	< 0.001
	Pizzas	480	510[a]	520[b]	500	460	450	424[a,b]	480	0.002	< 0.001
	Salads	247	264	261	245	278	280	295	270	0.798	0.174
**Nutrient per serve**											
Energy (kJ)	Ready meals	1558	1558	1610	1629	1621	1670	1649	1620	0.074	
	Pizzas	1056	1103	1065	1070	1132	1150	1041	1089	0.617	
	Salads	641	815	882	774	917	816	857	842	0.182	
Protein (g)	Ready meals	17.9[a,b]	18[c]	18.2[d]	18.6	18.9	20.1[a,c,d]	19.2[b]	18.8	< 0.001	
	Pizzas	11.4	11.4	11.1	11.4	12.0	12.1	10.0	11.3	0.654	
	Salads	4.3[a]	5.3	7.3[a,b]	7.2	5.9	4.9[b]	5.5	5.5	0.008	
Total fat (g)	Ready meals	10.5	10.7	11.5	10.5[a]	11.7	12.6[a]	12.7	11.8	0.004	
	Pizzas	8.4	9.6	8.6	8.5	7.5	8.6	7.6	8.3	0.899	
	Salads	10.2	11.3	10.8	10.4	11.9	11.3	10.0	10.7	0.540	
Saturated fat (g)	Ready meals	4.6	4.6	4.9	4.2	4.8	4.9	4.8	4.8	0.493	
	Pizzas	4.1	4.1	4.0	3.6	3.8	4.0	3.3	3.8	0.735	
	Salads	1.3	1.4	1.5	1.4	1.5	1.7	1.4	1.5	0.646	
Carbohydrate (g)	Ready meals	46.7	45.2	43.4	44.6	44.5	43.4	44.4	44.4	0.412	
	Pizzas	31.0	30.3	29.9	29.5	35.4	33.6	33.2	31.5	0.062	
	Salads	12.5	15.3	15.7	14.9	16.2	13.3	14.7	15.0	0.381	
Sugars (g)	Ready meals	6.7	7.5	6.8	7.0	6.7	7.0	6.9	6.9	0.634	
	Pizzas	4.9	5.3	4.3	4.3	3.8	4.8	5.2	4.7	0.131	
	Salads	4.8	5.0	3.7	3.8	5.1	5.4	5.1	4.8	0.059	
Fibre (g)	Ready meals	5.1	4.5	4.8	4.8	4.8	5.2	4.9	4.8	0.378	
	Pizzas	1.6	3.3	2.5	2.8	2.6	2.0	2.4	2.3	0.073	
	Salads	2.9	3.2	3.4	2.9	3.1	2.9	3.1	3.1	0.139	

Sodium (mg)	Ready meals	875[a,b]	900[c,d,e,f,g]	830[c]	812[a,d]	833[e]	792[a,f]	784[b,g]	816	< 0.001
	Pizzas	476	506	506	504	481	550	472	500	0.872
	Salads	332	366	358	338	375	359	387	362	0.782
Fibre displayed (n, %)	Ready meals	52 (21.4)	102 (42.3)	243 (63.3)	251 (61.8)	235 (60.4)	270 (60.0)	314 (67.1)	1467 (56.8)	
	Pizzas	8 (13.3)	12 (22.2)	25 (27.2)	31 (29.0)	23 (29.1)	25 (25.3)	28 (28.6)	152 (25.8)	
	Salads	21 (53.8)	34 (70.8)	76 (80.0)	67 (81.7)	92 (82.1)	97 (96.0)	116 (87.2)	503 (82.5)	

Data for 2014 to 2019 from FoodTrack™ database; †ALDI products introduced in 2016 and IGA products introduced in 2017; ‡Data for 2020 from Woolworths, Coles and ALDI in Adelaide; §Kruskal-Wallis tests; Values for years within a row with like superscript letters significantly different using the Bonferroni post hoc test ($p = 0.05$); *Jonckheere Trend tests.

Table S4: Health Star Rating of ready meals, pizzas and salads displaying the rating from 2014 to 2020.

Health Star Rating	Type of product	Year 2014 n	%	2015 n	%	2016† n	%	2017† n	%	2018 n	%	2019 n	%	2020‡ n	%	Total n	%
2	Ready meals	0	0.0	1	1.7	0	0.0	0	0.0	1	0.4	3	1.1	3	1.0	8	0.6
	Pizzas	0	0.0	1	25.0	4	18.2	7	17.9	8	15.1	8	12.9	8	11.6	36	14.5
	Salads	0	0.0	0	0.0	0	0.0	0	0.0	0	0.0	0	0.0	0	0.0	0	0.0
2.5	Ready meals	0	0.0	0	0.0	1	0.5	3	1.3	3	1.3	3	1.1	5	1.7	15	1.2
	Pizzas	0	0.0	2	50.0	3	13.6	2	5.1	4	7.5	3	4.8	1	1.4	15	6.0
	Salads	0	0.0	0	0.0	0	0.0	0	0.0	0	0.0	0	0.0	0	0.0	0	0.0
3	Ready meals	2	100.0	11	19.0	26	13.1	34	14.7	34	14.7	36	13.6	45	15.0	188	14.6
	Pizzas	0	0.0	0	0.0	6	27.3	10	25.6	17	32.1	31	50.0	33	47.8	97	39.0
	Salads	0	0.0	4	13.8	4	7.4	7	11.1	10	11.5	12	12.5	12	11.3	49	11.1
3.5	Ready meals	0	0.0	33	56.9	115	57.8	132	56.9	143	61.6	152	57.4	172	57.3	747	58.0
	Pizzas	0	0.0	1	25.0	9	40.9	20	51.3	24	45.3	19	30.6	26	37.7	99	39.8
	Salads	3	42.9	13	44.8	28	51.9	33	52.4	42	48.3	42	43.8	33	31.1	194	43.9
4	Ready meals	0	0.0	12	20.7	52	26.1	61	26.3	48	20.7	67	25.3	68	22.7	308	23.9
	Pizzas	0	0.0	0	0.0	0	0.0	0	0.0	0	0.0	1	1.6	1	1.4	2	0.8
	Salads	3	42.9	11	37.9	17	31.5	18	28.6	26	29.9	38	39.6	53	50.0	166	37.6
4.5	Ready meals	0	0.0	1	1.7	5	2.5	2	0.9	3	1.3	4	1.5	6	2.0	21	1.6
	Pizzas	0	0.0	0	0.0	0	0.0	0	0.0	0	0.0	0	0.0	0	0.0	0	0.0
	Salads	1	14.3	0	0.0	5	9.3	5	7.9	8	9.2	4	4.2	8	4.5	31	7.0
5	Ready meals	0	0.0	0	0.0	0	0.0	0	0.0	0	0.0	0	0.0	1	0.3	1	0.1
	Pizzas	0	0.0	0	0.0	0	0.0	0	0.0	0	0.0	0	0.0	0	0.0	0	0.0
	Salads	0	0.0	1	3.4	0	0.0	0	0.0	1	1.1	0	0.0	0	0.0	2	0.5

Data for 2014 to 2019 from FoodTrack™ database; No products displayed a Health Star Rating < 2; †ALDI products introduced in 2016 and IGA products introduced in 2017; ‡Data for 2020 from Woolworths, Coles and ALDI in Adelaide.
